# The immune responses to different *Uropathogens* call individual interventions for bladder infection

**DOI:** 10.3389/fimmu.2022.953354

**Published:** 2022-08-23

**Authors:** Linlong Li, Yangyang Li, Jiali Yang, Xiang Xie, Huan Chen

**Affiliations:** ^1^ The School of Basic Medical Sciences, Southwest Medical University, Luzhou, China; ^2^ Public Center of Experimental Technology, Model Animal and Human Disease Research of Luzhou Key Laboratory, Southwest Medical University, Luzhou, China; ^3^ Nucleic Acid Medicine of Luzhou Key Laboratory, Southwest Medical University, Luzhou, China

**Keywords:** bladder infection, uropathogens, immune responses, individual intervention, uropathogen escherichia coli

## Abstract

Urinary tract infection (UTI) caused by uropathogens is the most common infectious disease and significantly affects all aspects of the quality of life of the patients. However, uropathogens are increasingly becoming antibiotic-resistant, which threatens the only effective treatment option available-antibiotic, resulting in higher medical costs, prolonged hospital stays, and increased mortality. Currently, people are turning their attention to the immune responses, hoping to find effective immunotherapeutic interventions which can be alternatives to the overuse of antibiotic drugs. Bladder infections are caused by the main nine uropathogens and the bladder executes different immune responses depending on the type of uropathogens. It is essential to understand the immune responses to diverse uropathogens in bladder infection for guiding the design and development of immunotherapeutic interventions. This review firstly sorts out and comparatively analyzes the immune responses to the main nine uropathogens in bladder infection, and summarizes their similarities and differences. Based on these immune responses, we innovatively propose that different microbial bladder infections should adopt corresponding immunomodulatory interventions, and the same immunomodulatory intervention can also be applied to diverse microbial infections if they share the same effective therapeutic targets.

## Introduction

Urinary tract infection (UTI) is the most common infectious disease of the urinary system caused by diverse uropathogens, affecting females and males of all ages (1). In 2019, the overall global incident cases of UTI were 4046.12 ✕ 10^5^, with 871.90 ✕ 10^5^ for males and 3174.22 ✕ 10^5^ for females (2). Notably, the incident cases of UTI increased by 60.40% in the past thirty decades. UTI results in dysuria, frequency, urgency, suprapubic pain, hematuria, and serious sequelae including frequent recurrences, pyelonephritis with sepsis, renal damage, and pre-term birth and significantly affects all aspects of the quality of life of the patients (3, 4). In addition, UTI ranges in severity from mild self-limitation to severe sepsis, with 20-40% mortality (2). UTI has been causing a huge burden on human health, medical resources, and financial expenditure (2). In the United States alone, UTI results in >10 million outpatient visits and $3.5 billion in societal costs per year (2, 5).

UTI is caused by main nine pathogens, epidemiologically covering almost 100% of UTI confirmed cases (1). These pathogens include *uropathogen escherichia coli* (UPEC), *Klebsiella pneumoniae* (*K. pneumoniae*), *Staphylococcus saprophyticus* (*S. saprophyticus*), *Enterococcus faecalis* (*E. faecalis*), *Group B Streptococcus* (GBS), *Proteus mirabilis* (*P. mirabilis*), *Pseudomonas aeruginosa* (*P. aeruginosa*), *Staphylococcus aureus* (*S. aureus*), and *Candida* spp. (*Candida.*) (1). Antibiotics are the first-line treatment options for UTI but the effectiveness is being increasingly limited due to the rise of bacterial resistance (6, 7) **(** **Table 1** **)**. More than 80% resistance of *Escherichia coli* (*E. coli*) isolated from UTI to amoxicillin-clavulanic acid, ciprofloxacin, and trimethoprim-sulfamethoxazole has been observed in developing countries (39). In developed countries such as the United States, the resistance of Enterobacteria to some antibiotics for UTI has exceeded 30% (39, 40). Both the World Health Organization (WHO) and the Infectious Disease Society of America (IDSA) claimed the lack of antibiotics for the main pathogens of UTI and urged countries around the world to develop new drugs and therapies that can replace the overuse of antibiotics (41, 42). Thus, people move their sights on the immune responses hoping to find some effective therapeutic targets to combat the infection (4, 43–45).

**Table 1:** Drug resistance and virulence factors of the main nine uropathogens.

	Drug resistance	Main virulence factors					Refs
		Adherence	Toxin	Immune evasion	Iron acquisition	Others
UPEC	Penicillin, tetracycline, vancomycin resistance is 100%, ampicillin resistance is 90%, and cefazolin, ceftriaxone, cefepime, levofloxacin, and ciprofloxacin resistance reaches 70% in China.	Type 1 pili Type 2 pili P pili Dr adhesion S pili F1C pili	HlyA Cnf1	Capsule	Aerobactin Enterobactin Salmochelin Yersiniabactin	Flagella	(8–10)
*K.m*	Ampicillin penicillin, tetracycline, vancomycin resistance is close to 100%, nitrofurantoin resistance exceeds 90%, and Cefpidoxime is close to 80% in China.	Type 1 pili Type 3 pili	Lps	Capsule	Aerobactin Enterobactin		(8, 9, 11–13)
*S.s*	Cefuroxime resistance is 81%, Ceftazidime resistance is 76%, Amoxicillin-Clavulanic Acid, Gentamicin resistance is more than 65% in Nigeria.	Aas adhesin Ssp adhesin SdrI adhesin Uaf adhesin	Aas			Urease	(9, 14–17)
*E.f*	The resistance to amikacin, gentamicin, cefuroxime, ciprofloxacin, and cotrimoxazole is close to 100% in Poland.	Ebp pili Esp pili Ace adhesin	Protease			SigV	(9, 18–21)
*GBS*	Tetracycline resistance is over 74%, erythromycin resistance is 63%, and the resistance to clindamycin and fluoroquinolones is over 40% in China.		βH/C	Capsule			(22–24)
*P.m*	Amoxicillin-clavulanat resistance is 100%, ampicillin and nitrofurantoin resistances are 75% in Nepal.	MR/P pili	HpmA HlyA Pta	Capsule ZapA	Proteobactin Yersiniabactin	Flagella Urease	(25–31)
*P.a*	Topiperacillin-tazobactam and ceftazidime resistances are 100%, cefepime resistance is 75% in Saudi Arabia.	Extracellular DNA Exopolysaccharides	ExoU ExoT Elastase Phospholipase Rhamnolipids	ExoS	Pyochelin Pyoverdi	QS	(32–35)
*Candida.*	Posaconazole resistance is 92% in Iran.	Als proteins	Phospholipase B				(36, 37)
*S.s*	Nitrofurantoin resistance is 100% in Poland.	ClfA and ClfB					(18, 38)

Aas: a hemagglutinin-autolysinadhesin, Als: agglutinin-like sequence, βH/C: β-hemolysin/cytolysin, *Candida.: Candida spp*, ClfA/B: Clumping Factors A and B, Cnf1: cytotoxic necrotizing factor 1, Ebp: endocarditis- and biofilm-associated, *E.f, Enterococcus faecalis*, Esp: enterococcal surface protein, ExoU/T/S: exoenzyme U/T/S, F1C pili: type 1-like immunological group C pili, *GBS, Group B streptococcus*, HlyA: α-hemolysin, Lps: lipopolysaccharide, HpmA: haemolysin, *K.p, Klebsiella pneumoniae*, MR/P pili: mannose-resistant Proteus-like, *P.a, Pseudomonas aeruginosa*, Pta: Proteus toxic agglutinin, *P.m, Proteus mirabilis*, P pili: pyelonephritis-associated pili, QS: Quorum sensing, *S.a, Staphylococcus aureus*, SdrI: a surface-associated collagen-binding protein, SigV: extracytoplasmic function sigma factor, *S.s, Staphylococcus saprophyticus*, SssF, S. saprophyticus surface protein F; Ssp: a surface-associated lipase, UafB: a cell wall-anchored protein, ZapA: an extracellular metalloprotease.

The bladder possesses a wide range of immune responses against diverse uropathogens, including inhibitors of adhesion and antimicrobial protein production (4, 43–45). The bladder immune responses to invading uropathogens have some in common but also show differences depending on the type of uropathogens. For example, both UPEC and GBS stimulate bladder epithelial cells (BECs) to produce the antimicrobial peptide LL-37, and it is surprising that LL-37 has antibacterial effects on UPEC, but promotes GBS infection in the bladder (46–48). As such, individual immunomodulatory intervention options for UTI should be taken based on immune responses to the specific uropathogen in the bladder. Improved understanding of the bladder immune responses to diverse uropathogens is crucial for our ability to design immunomodulatory interventions and target them properly.

In this Review, we comparatively analyzed the similar and different immune responses triggered by the main nine uropathogens in the bladder. Based on the immune responses, we discussed the immune therapeutic targets with great prospects in-depth and innovatively proposed that when the bladder infection is treated through the modulation of immune responses, different uropathogens should adopt corresponding modulation options to improve the therapeutic effects.

## The bladder immune responses to the main nine uropathogens

Since the differences in virulence factors of the nine uropathogens **(** **Table 1** **)**, the immune responses against the nine uropathogens are diverse in the bladder. In this section, we summarize the characteristics and research status of immune responses to the major nine uropathogens in bladder infection.

### UPEC

**Figure 1:**
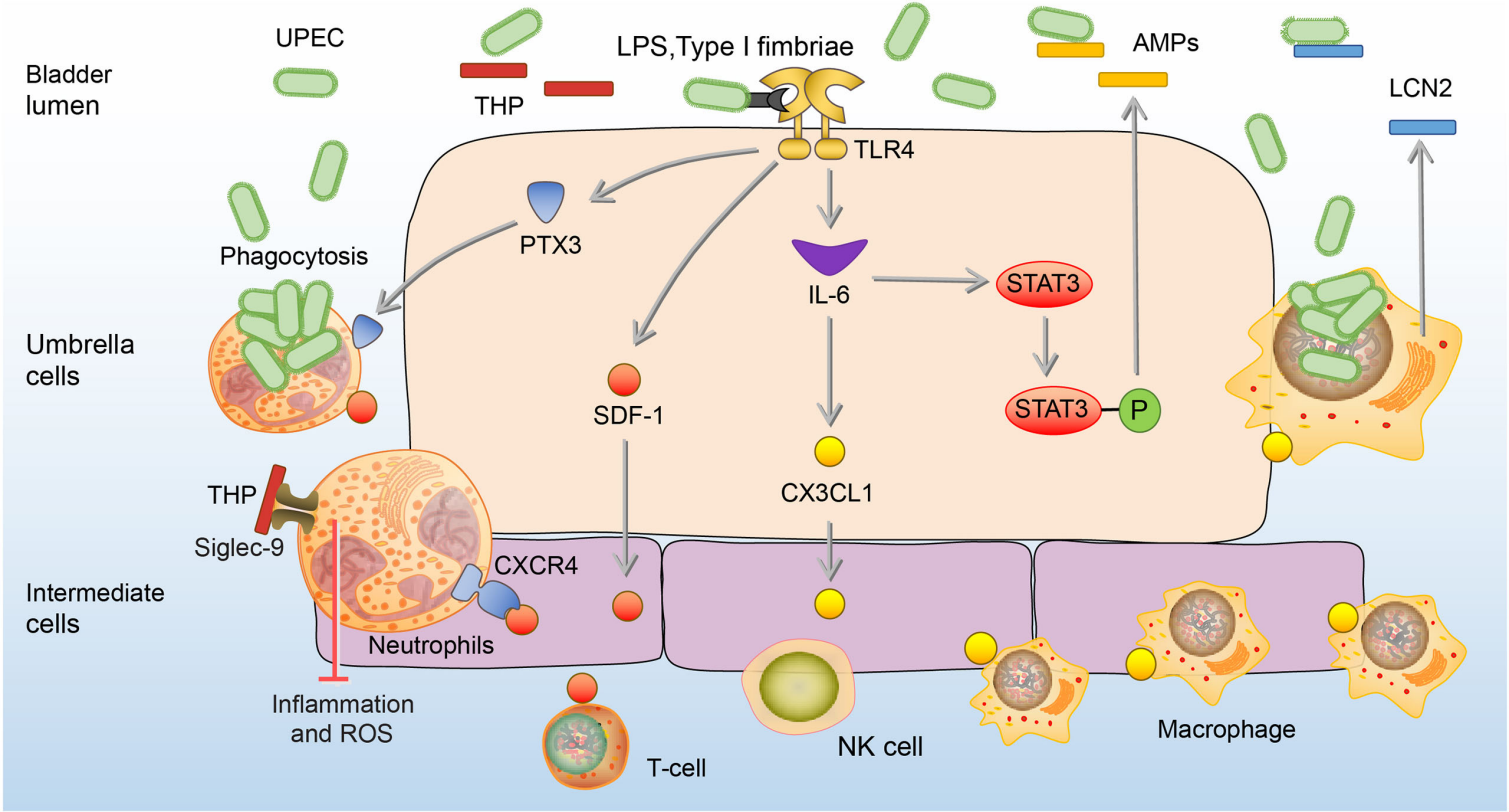
Extracellular immune responses to UPEC in the bladder. At the beginning of infection, THP reduces the adhesion of UPEC to the BECs. In addition, the THP can prevent excessive inflammation and ROS production of neutrophils. Once adhesion, BECs secrete SDF-1, PTX3, and IL-6. SDF-1 recruit neutrophils, T-cells, and NK cells to the site of infection. PTX3 promotes neutrophils to engulf UPEC, and IL-6 promotes the expression of CX3CL1 to recruit macrophages which kill UPEC by phagocytosis and LCN2. IL-6 also enhances the release of AMPs through phosphorylation of Stat3.AMPs, antimicrobial peptides; BEC, bladder epithelial cells; CXCR4, CXC-motif chemokine receptor 4; CX3CL1, C-X3-C motif chemokine 1; LCN2, lipocalin-2; IL-6, interleukin 6; NK cells, natural killer cells; PTX3, Pentraxins; SDF-1, stromal cell-derived factor1; Siglec-9, sialic acid-binding Ig-like lectin-9; Stat3, signal transducers and activators of transcription 3; THP, Tamm-Horsfall protein; UPEC, *Uropathogenic Escherichia coli*.

UPEC is the most common uropathogen of bladder infection (49). When UPEC ascends to the bladder along the urinary tract, it adheres to the mannose receptors of BECs through type I fimbriae (50). Tamm-Horsfall glycoprotein (THP), the most abundant urine protein, plays a key role to prevent the adhesion of UPEC to the BECs (51, 52). THP has a high-mannose structure among its disaccharides, which binds to the type I fimbriae and competes with the mannose receptors of BECs, thereby reducing the adhesion and colonization of UPEC to the bladder, and leading to the elimination of UPEC through urination (53, 54). In addition, the THP can prevent excessive inflammation in bladder infection *via* inhibition of the chemotaxis and reactive oxygen species (ROS) production by binding to sialic acid-binding Ig-like lectin-9 (Siglec-9) receptor of the neutrophils (55). Once UPEC successfully adheres to BECs, extracellular immune responses will be activated by lipopolysaccharide (LPS) and type I fimbriae of UPEC *via* binding to toll-like receptor 4 (TLR4) on BECs (56). The activation of TLR4 stimulates BECs to secrete stromal-cell derived factor 1 (SDF-1), and interleukin- 6 (IL-6) (57, 58). SDF-1 can bind to the CXC-motif chemokine receptor 4 (CXCR4) on neutrophils and recruit them to accumulate to the infection site (57). The aggregated neutrophils have the ability to engulf UPEC and can be significantly enhanced by BECs-secreted pentraxins (PTX3) (59). Cytokine IL-6 upon activation of TLR4 promotes the expression of C-X3-C motif chemokine 1 (CX3CL1) and recruits macrophages to the epithelium, which kill UPEC by phagocytosis and lipocalin-2 (LCN2) (60). LCN2 can restrict access of UPEC to iron, one of the key nutrients for the growth of UPEC, and starve them to death (61). Besides, IL-6 can enhance the expression of antimicrobial peptides (AMPs), such as ribonuclease 7 (RNase 7) and LL-37, which exert antibacterial effects by disrupting the microbial membrane (47, 58, 62–64). In the bladder of mice lacking RNase 7 and LL-37, the UPEC communities are significantly increased (47, 63). **(** **Figure 1** **)**

**Figure 2:**
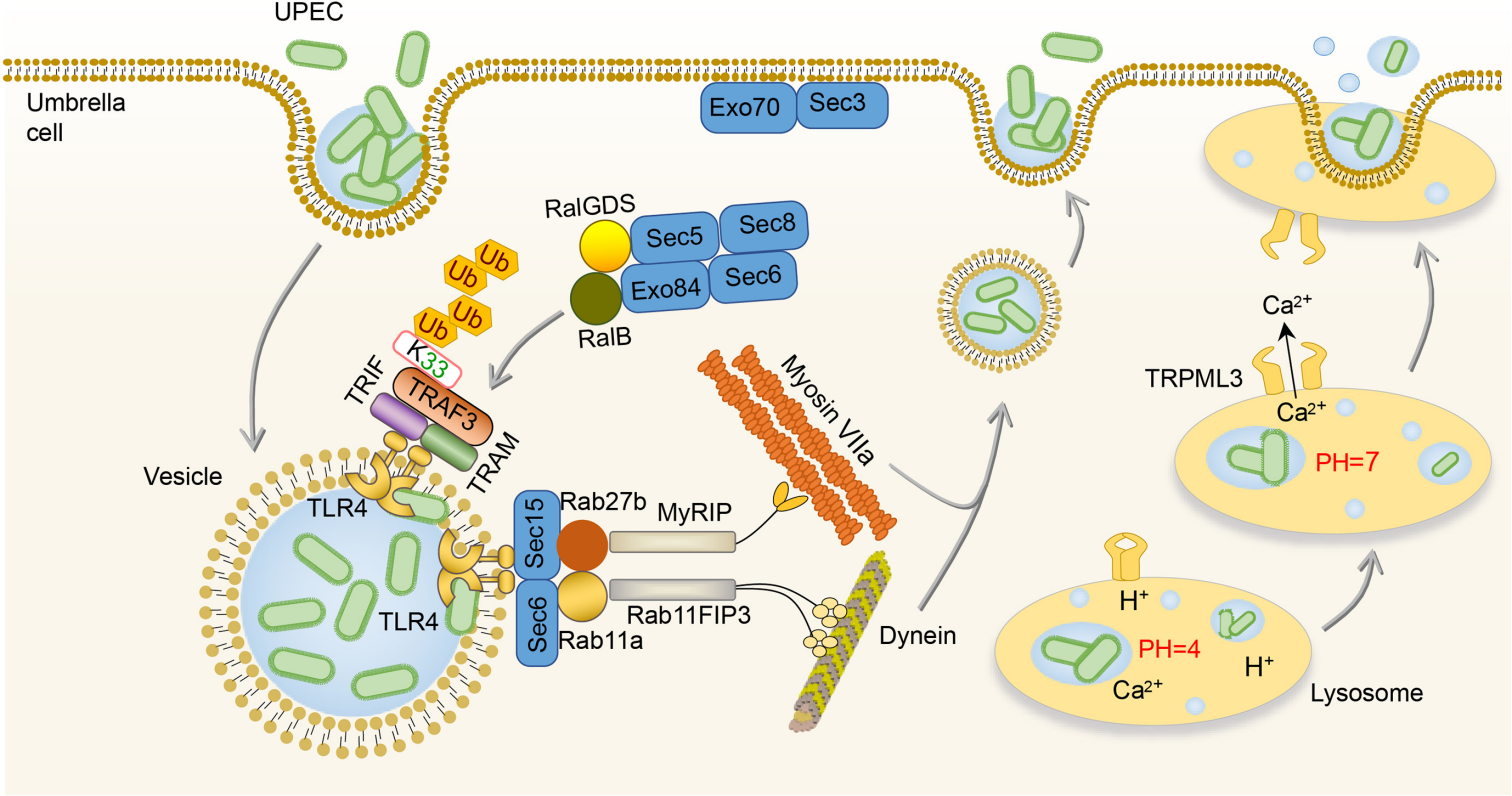
Intracellular immune responses to UPEC in the bladder. After invading BECs, TLR4 is activated by UPEC to promote the K33-linked polyubiquitination of TRAF3, which is sensed by the RalGDS-activating exocyst complex to locate and tether vesicles. Then, the Sec 6 and Sec 15 of the exocyst complex stimulate collaboration between Rab11a/Rab11FIP3/Dynein and Rab27b/MyRIP/MyosinVIIa to transport UPEC-containing vesicles. Once the lysosome engulfs UPEC, TRPML3 senses the pH neutralization and then releases calcium ions, leading to the efflux of UPEC. BEC, bladder epithelial cells; TLR4, toll-like receptor 4; TRPML3, transient receptor potential 3; UPEC, *uropathogenic Escherichia coli*.

Some UPEC survives from the extracellular immune responses and invades BECs, which then initiate the intracellular efflux immune responses (65, 66). Once BECs are invaded, two waves of UPEC expulsion in an innate immune signaling-orchestrated process occur (67). The first wave is mediated by the activation of TLR4 between 4 and 6h after infection followed by the second mucolipin transient receptor potential 3 (TRPML3)-activated wave occurring around 8h after infection (67). In the first wave of UPEC expulsion, UPEC is encapsulated within RAB27b^+^ vesicle and activates TLR4 by type I fimbriae (67, 68). Activation of TLR4 signaling advances the K33-linked polyubiquitination of TNF receptor associated factors (TRAF3), which is then sensed by the RalGDS-activating exocyst complex to locate and tether vesicles (68). After that, Sec 6 and Sec 15, two submit of the activated exocyst complex, stimulate collaboration between Rab11a/Rab11FIP3/Dynein and Rab27b/MyRIP/MyosinVIIa to transport UPEC-containing vesicles (67, 69). In addition, the activation of TLR4 can lead to the increase of cyclic adenosine monophosphate (cAMP) which subsequently stimulates the caveolin-1/Rab27b/PKA/MyRIP complex formation, and as a consequence, expels UPEC from infected BECs (70). Once UPEC escapes the first wave of efflux immune response by destroying the RAB27b^+^ vesicle, the second wave is initiated by lysosomal autophagy (71). After the lysosome engulfed UPEC, the pH of the lysosome will change from acid to neutral, and TRPML3 is able to sense the UPEC-mediated lysosome neutralization of pH and release calcium ions, which leads to the efflux of UPEC (71). **(** **Figure 2** **)**

**Figure 3:**
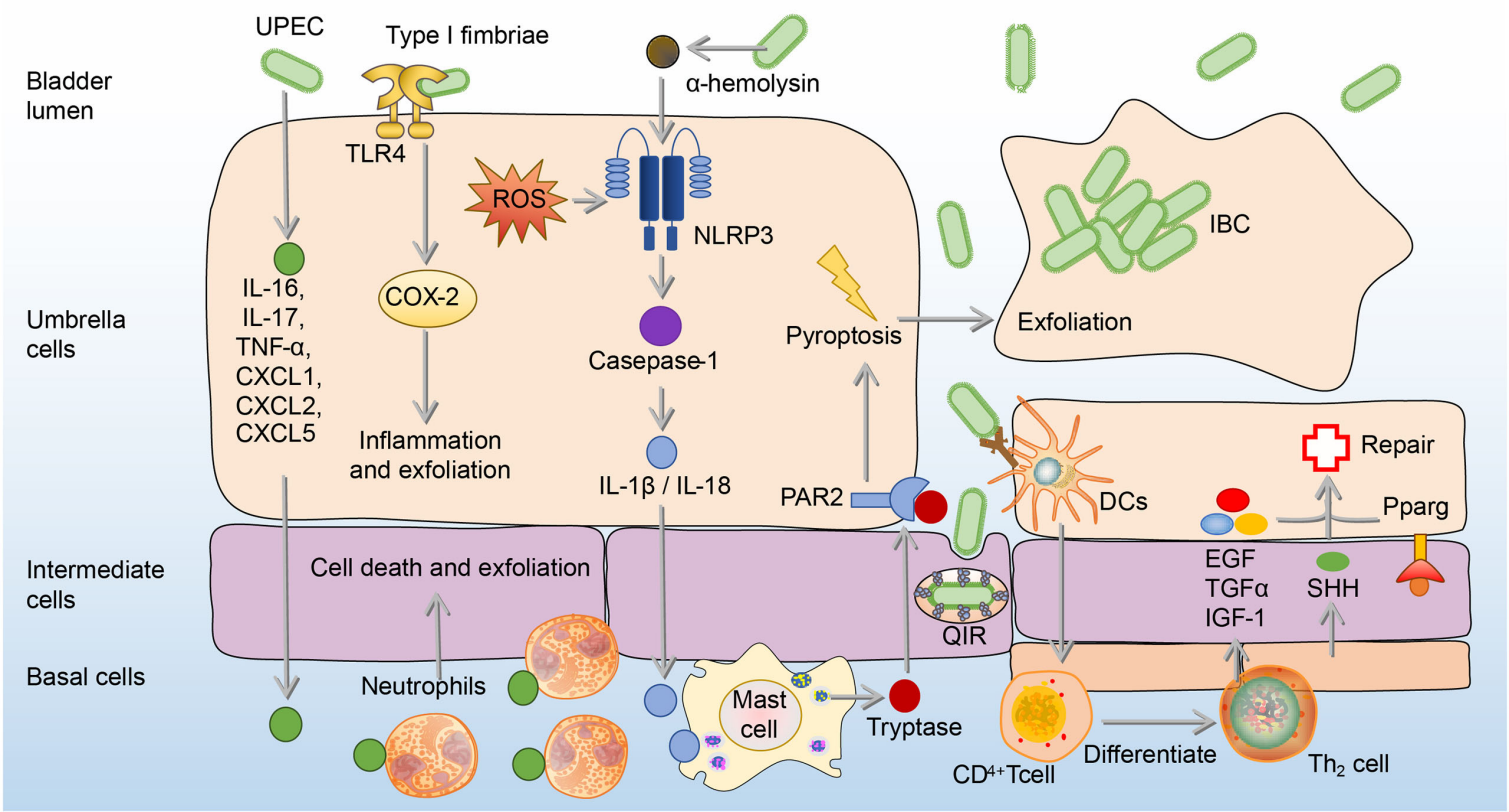
The exfoliation and regeneration of BECs in UPEC bladder infection. Cytokines from BECs are released to recruit neutrophils to induce cell death and exfoliation. Besides, Type 1 fimbriated UPEC activates TLR4 and causes the expression of COX-2, which promotes inflammation and exfoliation of BECs. Moreover, α-hemolysin produced by UPEC recruits mast cells through the ROS/NLRP3/caspase-1/IL-1β, which produces tryptase to mediate the BECs exfoliation. To repair shed BECs, transitional BECs will regenerate under the influence of EGF, TGF-α, IGF-1, SHH, and Pparg. BECs, bladder epithelial cells; EGF, epidermal growth factor; ERK, extracellular signal-related kinase; IGF-1, insulin-like growth factors-1; IL-1β, interleukin 1β; JNK, c-Jun-NH2-terminal kinase; NLRP3, NOD-like receptor thermal protein domain associated protein 3; PAR2, Protease-activated receptor 2; Pparg, peroxisome proliferator-activated receptor-γ; ROS, reactive oxygen species; SHH, sonic hedgehog; TGF-α, transforming growth factor-α; UPEC, *uropathogenic Escherichia coli*.

BECs can adopt more intense immune responses against UPEC by secretion of IL-6, IL-17, tumor necrosis factor-α (TNF-α), C-X-C motif chemokine ligand 1 (CXCL1), CXCL2, and CXCL5, which result in extensive neutrophil recruitment to induction of BECs’ death and exfoliation (72–75). BECs’ death and exfoliation carry a large amount of UPEC into the urine and then excretes UPEC by urination (72–75). In addition, in response to α-hemolysin, which is a virulence factor expressed by UPEC, human BECs induce the production of IL-1β and IL-18 through p38/ERK/ROS/NLRP3/caspase-1 signaling to recruit mast cells, which can produce tryptase to promote the exfoliation of BECs (76, 77). A point worthy of attention is that ROS and inflammation associated with NOD-like receptor thermal protein domain associated protein 3 (NLRP3) or cyclooxygenase-2 (COX-2) also contribute to BECs’ exfoliation (76–79). However, excessive ROS and inflammation are believed to do more harm than good to the host, since the bladder infection gradually intensifies with the increase of ROS and inflammation (80, 81). Although the exfoliation of BECs promotes the excretion of UPEC into the urine, it also exposes deep immature epithelium, thus allowing UPEC to invade them and form quiescent intracellular reservoirs (QIRs), which can avoid immune responses and antibiotics (82). In order to prevent the formation of QIRs caused by shedding, the proliferation ability of the epithelial layer after shedding is enhanced (83). This ability is mainly related to Th2 cells, as Th2 cells have an ability to secret epidermal growth factor (EGF), transforming growth factor-α (TGFα), and insulin-like growth factors-1 (IGF-1), which contribute to epithelial regeneration (84). The differentiation of Th2 cells in the bladder mainly depends on dendritic cells (DCs) presenting UPEC antigen to CD4+ T cells after infection (84). In addition, sonic hedgehog (SHH) expressed by basal stem cells and peroxisome proliferator-activated receptor-γ (Pparg) expressed by BECs also contribute to the regeneration and proliferation of BECs (85–87). **(** **Figure 3** **)**

### K.pneumoniae

**Figure 4:**
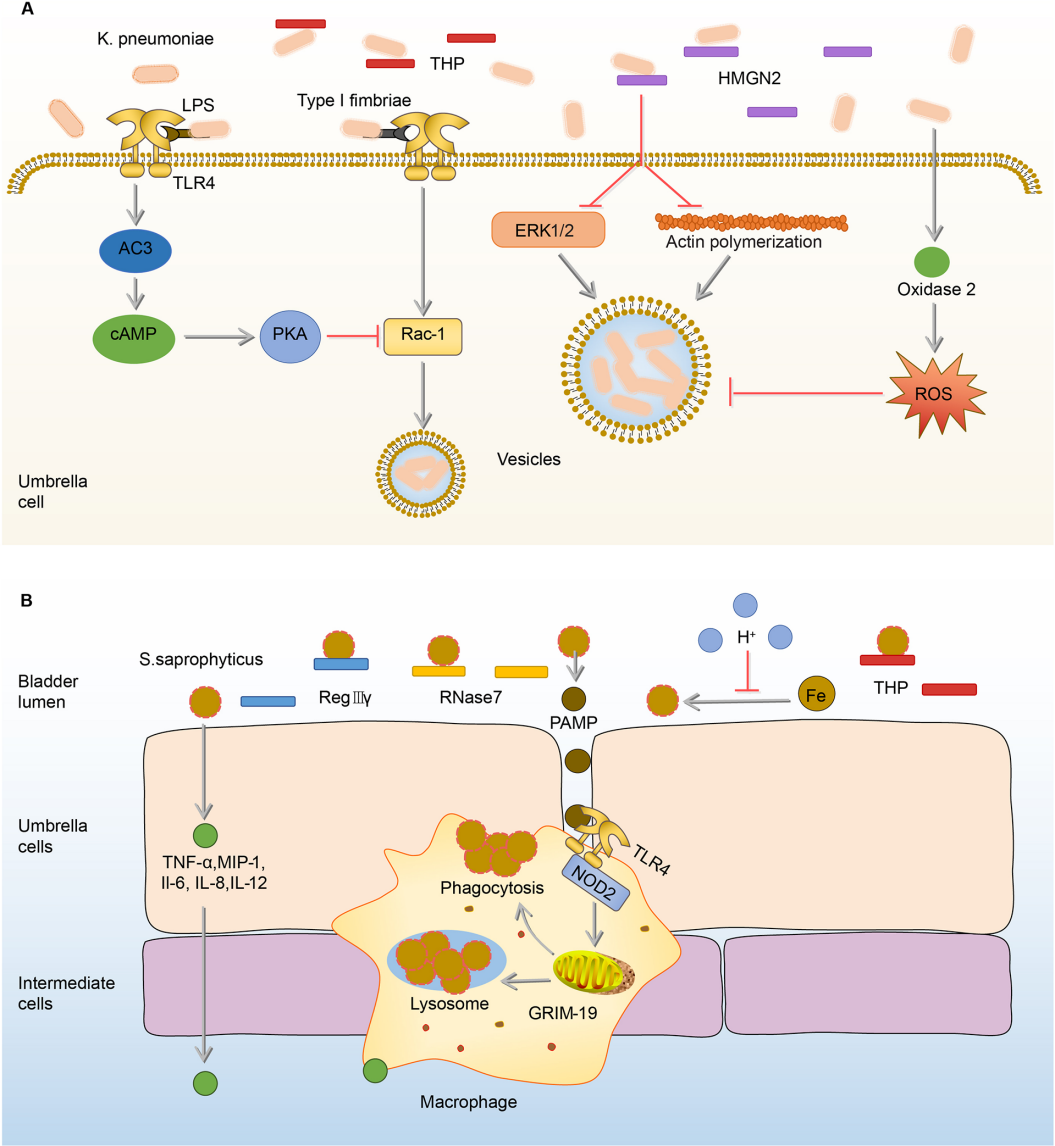
Immune responses to K. *pneumoniae* and *S.saprophyticus* in the bladder. **(A)** In the urine, THP exerts anti-adhesion and anti-inflammation effects on K. *pneumoniae*. Once adhered, K. *pneumoniae* lipopolysaccharide activates TLR4 to initiate AC-3/cAMP/PKA signaling pathway, then down-regulates Rac-1 and abrogates the endocytic lipid raft. HMGN2 also can inhibit K. *pneumoniae* internalization by inhibiting the attachment of bacteria and reducing bacterial-induced ERK1/2 activation and actin polymerization. In addition, the ROS promoted by oxidase 2 can inhibit endocytosis. **(B)** Before adhesion, RegIIIγ, RNase 7, and THP have anti-adhesion and sterilization abilities to *S. saprophyticus*. The acidic urine environment suppresses *S. saprophyticu* uptake and utilization of iron thus limiting its growth. After the adhesion, BECs produce TNF-α, MIP-1, IL-1, IL-6, and IL-12 to recruit macrophages. Upon the activation of TLR4 by PAMP, macrophages phagocytize *S.saprophyticus* depending on genes associated with GRIM-19. AC-3, adenylyl cyclase-3; cAMP, cyclic adenosine monophosphate; ERK1/2, extracellular-regulated kinase 1/2; GRIM-19, genes associated with retinoid-IFN-induced mortality-19; HMGN2, high-mobility group protein N2; IL-1, interleukin-1; INF-γ, interferon-γ; *K.pneumoniae*, *Klebsiella pneumoniae*; MIP-1, macrophage inflammatory protein-1; PAMP, pathogen-associated molecular pattern; PKA, protein kinase A; RegIIIγ, regenerating islet-derived 3γ; RNase 7, ribonuclease 7; ROS, reactive oxygen species; *S.saprophyticus*, *Staphylococcus saprophytes*; THP, Tamm-Horsfall protein; TLR4, toll-like receptor 4; TNF-α, tumor necrosis factor-α.

*K. pneumoniae*, one of the most common pathogens of intensive care unit infections, is the second leading cause of UTI from community or hospital sources (1, 88–90). Similar to the effects of THP on UPEC, THP exerts anti-adhesion and anti-inflammation effects on *K. pneumoniae* (91). In the THP-deficient mouse models, *K. pneumoniae* load in the urine and bladder significantly increased, as well as the number of inflammatory cells (91, 92). Once *K.pneumoniae* adheres to and invades BECs, intracellular immune defense mechanisms are initiated to inhibit the internalization of *K.pneumoniae* and promote its efflux. The first mechanism is initiated by TLR4, which down-regulates Rho through the expression of cAMP, and ultimately achieves the goal of inhibiting the invasion of *K.pneumoniae* (92). The second mechanism is mediated by high-mobility group protein N2 (HMGN2), which plays a key role in the inhibition of *K.pneumoniae* internalization by reduction of bacteria-induced activation of extracellular signal-regulated kinase (ERK1/2) and the polymerization of actin (93, 94). The last mechanism is that the invasion of *K.pneumoniae* promotes the synthesis of dual oxidase 2, which has the ability to inhibit bacterial internalization by the production of intracellular ROS (95, 96). The proper concentration of ROS has antibacterial against invading pathogenic bacteria (95, 97–99). **(** **Figure 4A** **)**

The type I fimbriae of *K.pneumoniae* is involved in the triggering of multiple immune responses in the bladder, which are very similar to UPEC type I fimbriae-induced immune responses (91, 92). Both UPEC and *K.pneumoniae* can be inhibited by the effect of THP against type I fimbriae, and they can both increase cAMP through type I fimbriae to regulate actin and ultimately promote bacterial efflux (53, 70, 91, 92). In addition, the UPEC and *K. pneumoniae* type I fimbriae play similar roles in the pathogenic process of bladder infection, as both of them rely on type I fimbriae to attach, invade, and form intracellular bacterial communities (1). By comparing the nucleic acid sequences of UPEC and *K.pneumoniae* type I fimbriae, they are highly homologous, which can explain why UPEC and *K. pneumoniae* type I fimbriae play similar roles in the pathogenicity and stimulate resembling immune responses of bladder infection (100, 101). However, *K.pneumoniae* carries the gene fimK but lacks the gene fimX, leading to reduce expression of type I fimbriae, which may explain *K. pneumoniae* form fewer intracellular bacterial communities (IBCs) and have lower titers in the bladder than UPEC and are more easily cleared by host defense response during infection (102).

### S.saprophyticus

Bladder infection caused by *S.saprophyticus* is most likely to occur in sexually active, non-pregnant women (103). Generally speaking, when *S.saprophyticus* contaminates the vaginal area, it ascends through the urinary tract (103). In the ascending process, *S.saprophyticus* uses citrate in urine to synthesize carboxylate siderophores and obtain iron ions in urine to supply its nutrition and growth (104). In order to limit the growth of *S.saprophyticus*, the bladder maintains a weakly acidic urine environment to reduce the activity of citrate synthase and thereby reduce the synthesis of citrate, ultimately achieving the goal of limiting *S.saprophyticus* from obtaining iron and starving them to death (104, 105). In addition, THP in urine has the ability to inhibit the adhesion of *S.saprophyticus* to BECs, which is similar to the effects on UPEC (53, 91). However, the antibacterial ability of urine is limited, as some *S.saprophyticus* still survive from THP and the acidic environment and adhere to BECs, stimulating BECs to increase the expression of AMPs including regenerating islet-derived 3γ (RegIIIγ) and RNase 7 (106, 107). RegIIIγ is able to promote the proliferation and repair of the injury epithelial cells (108, 109). RNase 7 mainly binds to the negatively charged bacterial cell membrane through cationic residues on its surface, destroys the physical and physiological functions of the bacteria, and ultimately kills the bacteria (62). In addition to AMPs, BECs mediate the production of cytokines, such as TNF-α, macrophage inflammatory protein-1 (MIP-1), IL-1, IL-6, and IL-12, to recruit the macrophages (14). Macrophages depend on genes associated with retinoid-IFN-induced mortality-19 (GRIM-19), a component of the mitochondrial respiratory chain, to phagocytize *S.saprophyticus* (110, 111). In GRIM-19-deficient macrophages, the expression of IL-1, IL-6, IL-12, interferon-γ (INF-γ) cytokines, and phagocytic ability are significantly reduced (110). **(** **Figure 4B** **)**

The immune responses to *S.saprophyticus* in bladder infection have differences from these to other uropathogens, as the urine pH and GRIM-19 have abilities to inhibit the growth of *S.saprophyticus* (104, 110). Acidic urine reduces the synthesis of citrate, consequently resulting in inhibition of *S.saprophyticus* growth, and GRIM-19 molecule exerts immune defense effects by regulating the phagocytic ability of macrophages in bladder infection (104, 110). Therefore, modulating urine pH and GRIM-19 is a promising target for *S.saprophyticus* UTI.

### E.faecalis

*E.faecalis* is one of the most resistant gram-positive bacteria in UTI, which has caused great trouble for clinical treatment (112). Current research on the immune responses to *E.faecalis* bladder infection are more about the responses of macrophages, DCs, and Natural killer (NK) cells (113, 114).

Under normal circumstances, activation of TLR2-Toll/interleukin-1 receptor (TIR) on macrophages can trigger the production of chemokines dependent on the NF-κB signaling pathway, and recruit immune cells in the bladder (115, 116). However, *E.faecalis* has a TIR domain-containing protein structure, which is similar to the TIR domain of TLR2 on macrophages (113, 117). Hence, the TIR domain-containing protein of *E.faecalis* (TcpF) has an ability to compete with the TIR domain of human TLR2 to form TLR dimers, thereby further eliminating downstream signals and ultimately inhibiting the immune responses of macrophages in the bladder (113, 117). Therefore, immune responses of macrophages to *E.faecalis* and UPEC co-infected in the bladder are significantly inhibited compared to the infection of UPEC alone, consequently promoting UPEC virulence during a mixed-species bladder infection (113, 118).

**Figure 5:**
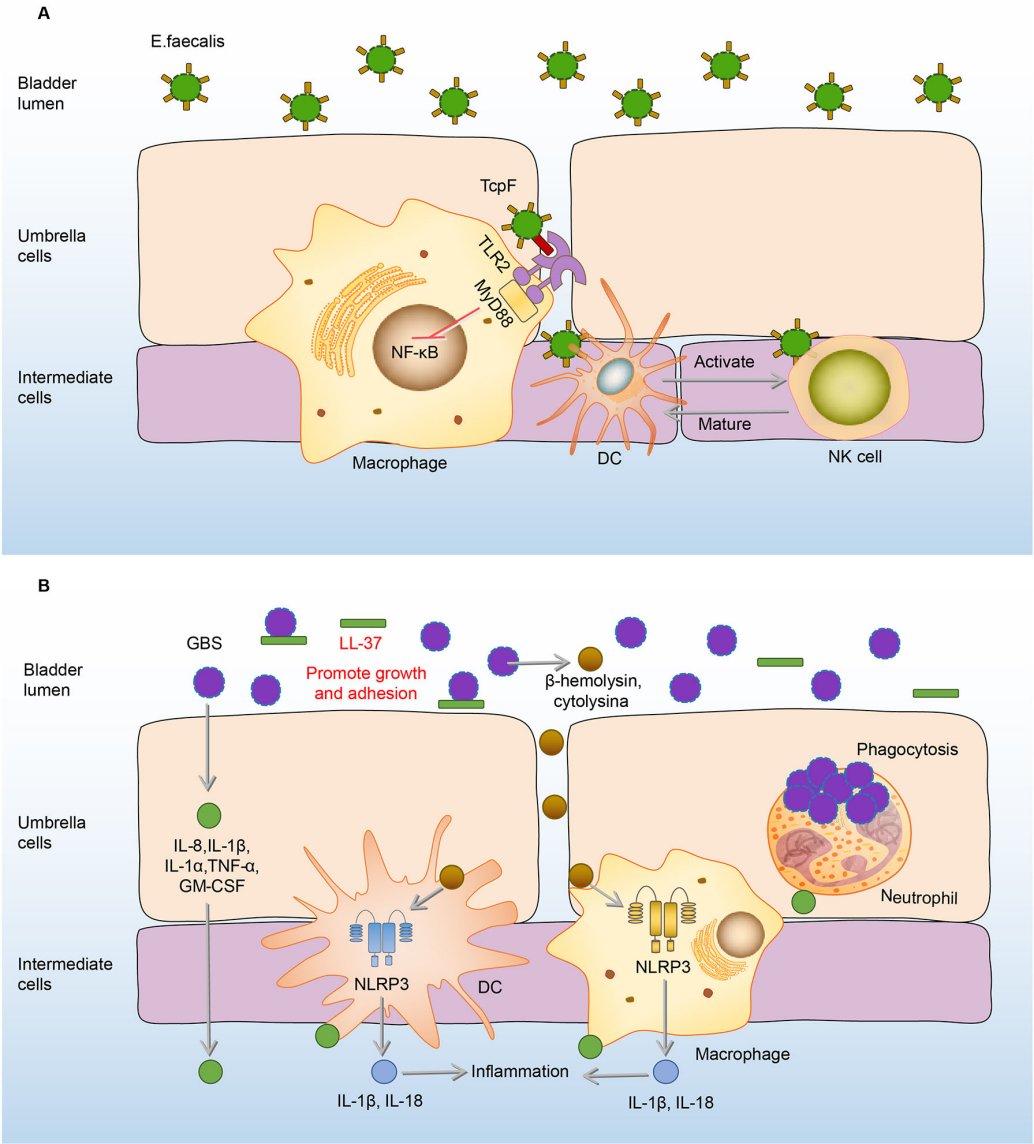
Immune responses to *E. faecalis* and GBS in the bladder. **(A)** Initially, RNase 7 in the urine binds to the *E. faecalis* and plays a bactericidal effect. Once *E. faecalis* adheres to BECs, TcpF of which binds to the TIR on macrophages, eliminating downstream MyD88 and NF-kB signals and suppressing the immune responses. However, the proliferation and activation of NK cells are intensified, which promote the maturation and differentiation of DCs. In turn, NK cells can be specifically activated to kill *E. faecalis* through derived effectors signals from infected DCs. **(B)** In the urine, LL-37 sticks to GBS and promotes its growth and adhesion. After adhesion, GBS induces the expression of IL-8, IL-1β, IL-1α, IL-6, TNF-α, GM-CSF to recruit immune cells and mediate inflammation. Macrophages and DCs secrete IL-1β and IL-18 against the GBS infection under the activation of the NLRP3 inflammasome by β-hemolysin/cytolysin of GBS. Neutrophils engulf GBS to play an antibacterial effect. DCs, dendritic cells; *E. faecalis*, *Enterococcus faecalis*; GBS, *Group B Streptococcus*; GM-CSF, granulocyte-macrophage colony-stimulating factor; IL-8, interleukin-8; MyD88, myeloiddifferentiationfactor88; NF-kB, Nuclear factor kappa beta; NK cells, natural killer cells; NLRP3, NOD-like receptor thermal protein domain associated protein 3; RNase 7, ribonuclease 7; TcpF, TLR2-Toll/Interleukin-1 receptor domain-containing protein of *E. Faecalis*.

Different from immunosuppressive effects on macrophages, *E.faecalis* has the ability to intensify the proliferation and activation of NK cells, which in turn promote the maturation and differentiation of DCs (114). In addition, NK cells also can be activated by *E.faecalis*-induced DC-derived effectors signals. *E. faecalis* specific DC/NK interaction is necessary for the killing of transformed or infected cells in *E.faecalis* bladder infection (114). **(** **Figure 5A** **)** The adaptive immune responses in the bladder are limited, widely assumed to the restricted ability of mature DCs to capture and present antigens in the bladder (119, 120). Exogenously regulating the DC/NK interaction may be one of the effective strategies to enhance bladder adaptive immune responses.

### GBS

GBS is a common commensal of the human genitourinary tract in healthy people (121). Nevertheless, this bacterium can cause life-threatening hazards to pregnant women, the elderly, and immunocompromised individuals (122–124).

When the immune function of the body is compromised, GBS in the urethra will express a variety of virulence factors to damage and adhere to the bladder tissue (122–124). AMPs in the urine are the first line of defense, however, LL-37, one of the AMPs, has no antibacterial effect on GBS (46). On the contrary, the load of GBS increases with the rise of LL-37 (46). Under the action of LL-37, GBS further adheres to the BECs, and this adherence promotes the expression of many cytokines, including IL-8, IL-1β, IL-1α, IL-6, TNF-α, granulocyte-macrophage colony-stimulating factor (GM-CSF) to mediate the occurrence of inflammation and recruit the immune cells including neutrophils, macrophages, and DCs to the infected sites (22, 125, 126). Neutrophils reach the focal point of infection producing anti-infective effects through various biological effects such as phagocytosis and cytokine production (125–128). Macrophages and DCs also make significant contributions to host defenses by secretion of IL-1β and IL-18 through the activation of the NLRP3 inflammasome, deficiency of which has GBS communities increased (129, 130). **(** **Figure 5B** **)** However, immune responses of neutrophils and macrophages can be inhibited by GBS virulence factors, as the cytokines production of macrophages and neutrophils increased when the bladder was infected by the virulence factor capsule sialic acid-deficient GBS (23, 131).

Compared with the anti-bacterial effects of LL-37 on UPEC infection, LL-37 plays an opposite role in GBS infection, which promotes GBS growth and proliferation (46, 47, 132). The role of NLRP3 may also differ between GBS and UPEC infection, as NLRP3-deficient mice were more susceptible to GBS infection and have GBS load increased. Whereas UPEC burden was significantly reduced in NLRP3-deficient BECs (76, 129). As these colonization differences between GBS and UPEC were observed based on the different NLRP3-deficient cells but have not been validated in the same cells and *in vivo* yet, which needs to be further explored (76, 129). Due to the differences in immune responses of the bladder between UPEC and GBS infection, when treating bladder infection caused by GBS, we should adopt different immunomodulation options from that of UPEC.

### P.mirabilis

**Figure 6:**
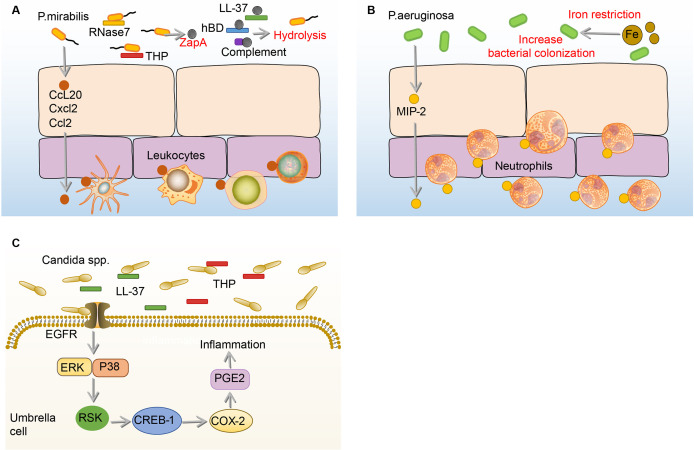
Immune responses to *P. mirabilis*, *Paeruginosa* and *Candida* spp. in the bladder. **(A)** Before adhesion, THP and RNase 7 resist the adhesion and invasion of *P.mirabilis* to the bladder, and *P.mirabilis* has countermeasures by expressing extracellular metalloprotease ZapA, which has hydrolytic activity. In addition, ZapA can hydrolyze complement (C1q and C3), LL-37, and human hBD in the urine. Notably, BECs can produce CCL20, CXCL2, and CCL2, and then promote numbers of leukocytes migrate to the epithelium, the specific role of which is not determined. **(B)** Under the iron restriction, *P.aeruginosa* has a stronger colonization ability on BECs. Once *P.aeruginosa* adheres to the BECs, the BECs increase the expression of MIP-1α to recruit neutrophils, which against the bladder infection of *P.aeruginosa*. **(C)** In the urine, THP and LL-37 respectively bind to the Als3 and Xog1p glycoprotein of *Candida* to inhibit adhesion. After *Candida* adhesion, BECs express COX-2 through EGFR-ERK/p38-RSK-CREB-1 pathway, leading to the synthesis of prostaglandins, which mediate the occurrence of inflammation. BECs, bladder epithelial cells; *Candida, Candida spp;* CCL20, c-c chemokine ligand 20; COX-2, cyclooxygenase-2; CREB-1, cAMP-response element-binding protein-1; CXCL2, C-X-C motif chemokine ligand 2; EGFR, epidermal growth factor receptor; ERK, extracellular regulated protein kinases; hBD, β-defensin; MIP-1α, macrophage inflammatory protein-1α; *P.aeruginosa*, *Pseudomonas aeruginosa*; *P. mirabilis*, *Proteus mirabilis*; RNase 7, ribonuclease 7; RSK, ribosomal s6 kinase; THP, tamm-horsfall protein.

*P.mirabilis*, which showed high resistance rates to ampicillin, nitrofurantoin, and amoxicillin-clavulanate, is the sixth most common pathogen of uncomplicated UTI (1, 25). When the *P.mirabilis* reaches the mouth of the urethra, it moves up the urethra through the swing of the flagella and reaches the bladder (133). During the ascending process, many immune mediators in the urine including complement (C1q and C3), LL-37, and human β-defensin (hBD) are hydrolyzed by ZapA (Mirabilysin), which is a 54-kDa extracellular proteolytic enzyme with broad-spectrum degradation activity encoded by *P.mirabilis* (26). Similar to the effects on UPEC and *K.pneumoniae*, THP and RNase 7 in the urine resist the adhesion and invasion of *P.mirabilis* to BECs (106, 134). Some *P.mirabilis* survive from THP and RNase 7 and adhere to BECs through fimbriae (135). Once the *P.mirabilis* successfully adhere, a number of leukocytes migrate to the epithelium mediated by the production of c-c chemokine ligand 20 (CCL20), CXCL2, and CCL2 under the stimulation of flagella (136). However, the migration of leukocytes is demonstrated ineffective in clearing *P.mirabilis* (136). **(** **Figure 6A** **)**

There are very few reports on the immune responses to the effective inhibition of *P.mirabilis* in bladder infection. Two broad-spectrum antibacterial mediators, THP and RNase 7, in the urine have been reported to inhibit the growth of *P.mirabilis* (106, 134). However, many immune responses and immune mediators in the urine are suppressed by ZapA (26). In addition, it has been reported that the anti-MrpA (structural subunit of MR/P fimbriae) antibodies in urine and serum can be neutralized by *P.mirabilis* (137). Therefore, the antibacterial immune responses to *P.mirabilis* in bladder infection remain lacking and need more to be explored in the future.

### P.aeruginosa

Of all uropathogens in bladder infection, *P.aeruginosa* is a relatively small pathogenic bacterium in UTI, but it has caused great trouble for clinical treatment, as many antibiotics such as topiperacillin-tazobactam, ceftazidime, and cefepime, which are effective against other uropathogens, hardly have effects on *P.aeruginosa* (32). The current research on the immune responses to *P.aeruginosa* in the bladder is extremely limited. Before *P.aeruginosa* adhere to the bladder, the growth of *P.aeruginosa* is firstly affected by iron restriction and THP (34, 138, 139). Surprisingly, the burden of *P.aeruginosa* and histopathological conditions in the bladder and kidney increase under iron-restricted conditions. Consistently, *in vitro* experiments showed that iron-restricted media increases the adhesion of *P.aeruginosa* to the BECs and inhibits macrophage to phagocytose *P.aeruginosa* (138). The reason why iron restriction can aggravate the *P.aeruginosa* bladder infection may be attributed to the enhancement of quorum sensing (QS) signaling molecules under iron deficiency conditions (140, 141). Furthermore, when mice are infected with THP-coated *P.aeruginosa*, the bacterial burden and pathological changes in the kidney are significantly enhanced (139). Therefore, THP and iron restriction have beneficial effects on *P.aeruginosa* colonization (34, 138, 139). Once the bladder is colonized by *P.aeruginosa*, it will increase the expression of MIP-1α to recruit neutrophils, which can effectively decrease the burden of *P.aeruginosa* in the bladder (142). **(** **Figure 6B** **)**

Many immune responses that have spectral antibacterial effects on other uropathogens have no effects on *P.aeruginosa*, or may even aggravate the infection of *P.aeruginosa*. In addition, many antibiotics, which are effective against other uropathogens, do not affect *P.aeruginosa* bladder infection (32). Hence, it is pretty urgent to continue to explore the effective immune defenses for *P.aeruginosa* in bladder infection so that propose some feasible immunomodulatory interventions.

### Candida.

*Candida.* is a common uropathogenic fungus in UTI, especially in immunocompromised patients (143). Generally speaking, *Candida.* mainly causes disease through its hyphae, *Candida.* adheres to the BECs through the agglutin-like sequence (Als3) glycoprotein structure on the hyphae in the bladder (144, 145). To combat this adhesion process, the THP already present in the bladder urine binds to Als3, thereby inhibiting the adhesion of *Candida.* to the BECs (144). In addition to THP, LL-37 binds to the Xog1p glycoprotein of the *Candida.* cell wall to reduce adhesion to BECs (146, 147). However, once *Candida.* adheres to the BECs, COX-2 will be induced in BECs through the EGFR-ERK/p38-RSK-CREB-1 pathway, the upregulation of which leads to the synthesis of prostaglandins, triggering inflammation (148, 149). **(** **Figure 6C** **)**

*Candida.* is the only fungus among the nine major uropathogens and the bladder executes different mechanisms of immune responses to *Candida.* from those to bacteria. For example, THP and LL-37 exert an anti-adhesion effect on both *Candida.* and other bacteria, THP targets the hyphae to inhibit the adhesion of *Candida (* 144–146 *).*. In bacterial infection, THP targets the fimbriae (52, 91, 134). LL-37 reduces adhesion of the *Candida.* by binding to its glycoprotein, in bacterial infection, LL-37 exerts anti-adhesion by disrupting the bacterial membrane (47, 146).

### S.aureus

*S.aureus* is the most common gram-positive bacteria in hospital-acquired infections, which mainly occur in catheter-induced UTI (150, 151). The immune responses of the bladder to *S.aureus* are blank. However, there are many patients with cystitis caused by *S.aureus*, which is highly resistant to antibiotics (1, 18, 152). It is necessary to carry out research work on the immune responses to *S.aureus* in bladder infection.

## Potential individual immunomodulatory interventions

Based on the above summarized immune responses to diverse uropathogens in bladder infection, we deemed that maybe an immune target has antibacterial effects on a variety of uropathogens in bladder infection, and on the other side, some immune mediators play opposite roles in bladder infection **(** **Table 2** **)**. In this section, we discuss the potential immunomodulatory interventions for bladder infection caused by different uropathogens.

**Table 2:** Potential immunomodulatory targets against different uropathogens in bladder infection.

Targets	THP	ROS	Iron restriction	AMPs				Hormones		cAMP	Urothelium repair	Anti-inflammation		Immunization with vaccines	Probitioc interventions
				RNase7	RegIIIγ	LCN2	LL-37	Insulin	estogen			COX-2	NLRP3		
UPEC	√		√	√		√	√	√	√	√	√	√	√	√	√
*K.p*	√	√	√					√		√				√	√
*S.s*	√		√	√	√										
*E.f*								√						√	√
GBS							×	√					×		
*P.m*	√		√	√										√	
*P.a*	×	√	×												√
*Candida.*	√						√	√				√			√
*S.a*								√							

“√” means that this immunomodulatory target has potential therapeutic value, and “×” means that this immunomodulatory target is not recommended; AMPs, antimicrobial peptides; Candida., Candida spp; COX-2, cyclooxygenase-2; *E.f, Enterococcus faecalis; GBS, Group B streptococcus; K.p, Klebsiella pneumoniae;* LCN2, lipocalin-2; NLRP3, nod-like receptor thermal protein domain associated protein 3; *P.a, Pseudomonas aeruginosa; P.m, Proteus mirabilis;* RegIIIγ, regenerating islet-derived 3γ; RNase 7, ribonuclease; ROS, reactive oxygen species; *S.a, Staphylococcus aureus; S.s, Staphylococcus saprophyticus;* THP, tamm-horsfall protein.

### Inhibition of adhesion

THP, a broad-spectrum anti-infective protein in bladder infection, has the ability to against many uropathogens, inclusive of UPEC, *K.pneumoniae*, *P.mirabilis*, *S.saprophyticus*, and *Candida* (91, 134, 144, 145, 153, 154). It plays the antibacterial effect mainly by reducing the colonization of uropathogens on BECs, as THP can occupy the binding sites of uropathogens to BECs (91, 134, 144, 145, 153, 154). Therefore, the upregulation of THP may be an excellent intervention option for the bladder infection caused by UPEC, *K.pneumoniae*, *P.mirabilis*, *S.saprophyticus*, and *Candida.* Clinical experiments showed that the level of THP in patients who take cranberry extract orally increases, and the urine from these patients has a stronger inhibitory effect on the adhesion of UPEC (52, 155). However, whether this intervention is effective for bladder infection caused by *P.aeruginosa* is not determined, as the THP can lead to an increase in *P.aeruginosa* load (139). In conclusion, the upregulation of THP is an excellent way to combat the bladder infection of UPEC, *K.pneumoniae*, *S.saprophyticus*, *P.mirabilis*, and *Candida.*

### Scavenging of ROS

Over accumulated ROS is involved in the induction of BECs injury and death in bladder infection, but the proper concentration of ROS has antibacterial effects (76, 77, 95, 97–99). Uropathogens including UPEC, *K.pneumoniae*, *S.saprophyticus*, *P.mirabilis*, *P.aeruginosa*, and *Candida.* induce an increase in ROS level in bladder infection (156). Reducing the expression of ROS seems to have a therapeutic effect on UTI (157, 158). The results of a systematic review showed that vitamin C, a drug candidate with antioxidant capacity, has the ability to prevent the occurrence of UTI, and anthocyanins can inhibit ROS to treat UTI caused by *K.pneumoniae* and *P.aeruginosa* (157, 158). Among the anthocyanin extracts of all plants, blueberry is an excellent candidate because of its very rich anthocyanin content (159, 160). We conclude that reducing the content of ROS through the use of antioxidant drugs is a promising intervention for bladder infection.

### Iron restriction

Iron restriction, as another broad-spectrum antibacterial method, inhibits the growth of a variety of uropathogens in bladder infection, including UPEC, *K.pneumoniae*, *S.saprophyticus*, and *P.mirabilis* (61, 104, 161–163). Exogenous regulation of iron content in urine is an excellent immune regulation target for the treatment of bladder infection. Animal experiments showed that the dietary restriction of iron significantly reduces the iron content, followed by bacterial burden, bacteriuria, as well as inflammatory responses decreasing in UPEC bladder infection, and the exogenous injection of lactoferrin, an iron-binding glycoprotein, also significantly reduces the UPEC load and the infiltration of neutrophils (164, 165). However, the intervention effects of iron restriction on UTI caused by *P.aeruginosa* are not verified, because iron restriction does not inhibit the growth of *P.aeruginosa*, but increases the bacterial load in the bladder (138). In conclusion, restricting access to iron is a promising intervention for bladder infection caused by UPEC, *K.pneumoniae*, *S.saprophyticus*, and *P.mirabilis*, which may not apply to *P.aeruginosa*.

### Increase of AMPs

AMPs are a large class of compounds that participate in a variety of innate immune responses and are considered to be promising compounds to deal with antimicrobial resistance (166). RNase 7 has antibacterial effects on UPEC, *S.saprophyticus*, and *P.mirabilis*, RegIIIγ has antibacterial effects on *S.saprophyticus*, LCN2 has antibacterial effects on UPEC, and LL-37 has antibacterial effects on UPEC and *Candida* (47, 58, 61, 106, 107, 147). Therefore, RNase 7, RegIIIγ, LCN2, and LL-37 may have therapeutic effects against the above uropathogens in bladder infection. Notably, different AMPs and even different segments of the same AMP have different antimicrobial effects. Taking RNase 7 as an example, fragments of RNase 7 have different antibacterial effects on uropathogens, the F:1-97 fragment has the most antibacterial activity against UPEC and *S.saprophyticus*, while all N-terminal fragments except the F:1-45 fragment have the most antibacterial activity against *P.mirabilis* (106). Notably, LL-37 does not have a killing effect on GBS, on the contrary, it will promote GBS bladder infection (46).

### Regulation of hormones

Among hormones, insulin has the ability to promote the secretion of RNase 7, RNase4, and LCN2, which are proven to be against bladder infection caused by a variety of uropathogens (167, 168). In addition, insulin reduces the risk factor of blood sugar, thereby reducing the susceptibility of diabetic patients to bladder infection of UPEC, *K.pneumoniae*, *E.faecalis*, GBS, *S.aureus*, and *Candida (* 124, 169–173 *).*. However, a prospective study showed that diabetic patients who used insulin for a long time had a higher risk of UTI than diabetic patients who did not use insulin. The reason for the inconsistency may be that the blood and urine sugar of patients taking insulin is higher than that of patients without taking insulin (174). Insulin may not be suitable for people with low blood sugar, because it can cause hypoglycemia and lead to undesirable consequences such as coma (175). Except for insulin, estrogen also changes the bacterial burden in bladder infection (176–178). Female, compared with male, had lower bacterial burdens and stronger immune responses (178). This may be because of the increase of IL-17 mediated by estrogen, as IL-17 initiates many anti-bacterial pathways, including antimicrobial peptide and chemokine expression and the direct killing effects on bacteria (178–181). Differently, exogenous androgen can increase the burden of UPEC and mediate the development of cystitis into pyelonephritis (176, 177).

### Enhancement of intracellular efflux bacteria

cAMP plays an important role in the efflux of UPEC and *K.pneumoniae* from BECs in bladder infection (70, 92). Many drugs, that are proven by US-Food and Drug Administration certification (like Liraglutide, Terbutaline, and so on) can increase the production of cAMP. Liraglutide, a glucagon-like peptide-1 (GLP-1) receptor agonist, is shown to increase cAMP to inhibit the replication of the hepatitis C virus (182). Terbutaline can reduce LPS-induced human pulmonary microvascular endothelial cell damage by increasing cAMP (183). cAMP is proven to be a potential immunomodulatory target for bacterial bladder infection, but there is a lack of research to prove their therapeutic effects, further research is needed (70).

### Urothelium repair

BECs play important roles as the first line of defense in bladder infection, because it produces many immune factors to mediate the immune responses, and meanwhile, it prevents the invasion of bacteria into the deep immature epithelium to form QIRs (184). Hyaluronic acid (HA), a high molecular weight glycosaminoglycan, not only induces the production of LCN2 and IL-8 in HA/flagellin-challenged epithelial cells but is also involved in the enhancement of the physical barrier of BECs (185). As clinical data showed that intravesical injection of HA can indeed achieve the purpose of treatment for infected humans (186–188). Similar to HA, clinical trials showed that 25-hydroxyvitamin D3 also has the role of protecting the bladder epithelial integrity in postmenopausal women, as 25-hydroxyvitamin D3 induces expression of occludin and claudin-14, which are the tight junction proteins in the urinary tract (189). In addition to protecting mature epithelial integrity, the measures to promote the regeneration of immature epithelium should be taken into consideration. Briefly, HA, 25-hydroxyvitamin D3 and so on which can repair urothelium are excellent targets to combat the infection of UPEC.

### Anti-inflammation

COX-2 and NLRP-3 were shown to favor infections by exacerbating inflammation (76–79, 148, 149, 190). Inhibiting the synthesis of COX-2 or NLRP-3 can protect mice from cystitis induced by uropathogens, but except GBS-induced cystitis, because GBS colonized more in NLRP-3-deficient mice compared with wild type mice (76–79, 129, 148, 149, 190). Therefore, inhibiting inflammation by targeting COX-2 or NLRP-3 theoretically has a certain therapeutic value against uropathogens except for GBS (129). However, a randomized controlled trial with a sample size of 253 showed that targeting COX-2 by using NSAIDs is less effective than antibiotics and may even promote the progression of cystitis to pyelonephritis (191). Another randomized controlled trial with a sample size of 383 also showed that NSAIDs are less effective than antibiotics in the treatment of bladder infections, and may even lead to pyelonephritis and serious adverse events (192). To sum up, although the basic experiments confirmed the value of anti-inflammatory in the intervention of bladder infection, it should be cautious in clinical application for UTI.

### Immunization with vaccines

Vaccination holds a promising approach against different microbial bladder infections. Many vaccines designed against individual-specific uropathogens are currently in the stage of basic or clinical trials (193–195). For UPEC bladder infection, there are vaccines targeting type 1 fimbriae, hemolysins, siderophore receptors, cytotoxic necrotizing factor 1 (CNF1), and LPS (194, 196–198). For *P.mirabilis* bladder infection, there are vaccines targeting MR/P fimbriae and hemolysins (199, 200). For *E. faecalis* bladder infection, there is endocarditis- and biofilm-associated (Ebp) fimbriae vaccine (201). To make the vaccines against the diversity of uropathogens, the vaccines can be extracted from a range of uropathogens to form a multivalent vaccine. For example, Urovac (Solco Basel Ltd, Basel, Switzerland) consists of 10 heat-killed uropathogens, including 6 serotypes of UPEC, *P.vulgaris*, *K.pneumoniae*, and *E.faecalis* (202). Although most vaccines have been demonstrated highly efficacious in reducing the incidence and severity of UTI in animal models, there is a lack of large-scale clinical trials to prove their efficacy and safety. As the purpose of vaccination is to induce immune memory of the specific pathogens, the vaccines are effective on the corresponding uropathogens but not on others. If a broad anti-infective effect is desired in the treatment of bladder infection, then a multivalent vaccine is an option.

### Probiotic interventions

Probiotics can inhibit the adherence, growth, and colonization of uropathogens and reduce inflammation in the urinary tract by producing antibacterial substances such as lactic acid and hydrogen peroxide, or by directly competing for the adhesion sites between UPEC and the BECs (203–205). The efficacy and safety of probiotics in the treatment of bladder infection have been confirmed by extensive clinical trials, which include *Lactobacillus rhamnosus*, *Lactobacillus acidophilus*, *Lactobacillus fermentum*, *Lactobacillus reuteri*, *Bifidobacterium bifidum*, and *Bifidobacterium lactis* (206–209). However, different probiotics were demonstrated to have diverse antibacterial effects. *Lactobacillus acidophilus* has an average inhibition zone of 16 mm for UPEC but for *E.faecalis* was 12mm (210). *Lactobacillus salivarius* UCM572 had anti-adhesion effects against UPEC, however, the anti-adhesion effect on other uropathogens was not demonstrated (211). Furthermore, the anti-adhesion effects of different *Lactobacillus* strains against *Candida*, *K.pneumoniae*, *P.aeruginosa*, and *Proteus* were reported to be different (212). Therefore, when probiotics are used to treat different microbial bladder infections, appropriate probiotic strains should be selected according to the specific uropathogens in bladder infection.

## Further research

Because of the diverse effects of immunomodulatory interventions on different uropathogens, corresponding immunotherapies should be taken for different uropathogenic bladder infections for better therapeutic effects. However, compared with great advances in the understanding of bladder immune responses trigged by UPEC, understanding of the bladder immune responses caused by other uropathogens remains relatively limited, which results in relatively few individual immunomodulatory options for other uropathogens which we came up with. Further research needs to pay more attention to the immune responses to other uropathogens besides UPEC. In addition, most of the immunomodulatory interventions were proven efficacious in animal models, further clinical research needs to demonstrate the consistency of the effects, and then which will achieve better therapeutic effects in the future.

## Conclusion

Antibiotic therapy is the only option for UTI treatment but in recent years it is becoming more limited due to the increasing resistance of UTIs to routinely applied antibiotics. Immunomodulatory interventions have been suggested to be alternatives. However, the bladder executes different immune responses depending on the type of uropathogens, thus one immunomodulatory target has diverse effects on different uropathogens. The similarities and differences in immune responses to the main nine uropathogens in bladder infection were sorted out and comparably analyzed in this Review. To improve the effects of immunomodulatory interventions on different microbial bladder infections, specific uropathogenic bladder infections should adopt corresponding immunomodulatory targets to intervene, and one immunomodulatory intervention can be applied to diverse microbial infections, under the condition that they share the same effective therapeutic targets. Only through individual treatments in different uropathogenic bladder infection by immunomodulatory interventions can achieve better therapeutic results as alternatives for antibiotics in the future.

## Author contributions

LL, YL, and HC researched data for the article and wrote the manuscript. HC and XX made substantial contributions to discussions of content, and reviewed and edited the manuscript. All authors contributed to multiple parts of the paper, as well as the final style. All authors contributed to the article and approved the submitted version.

## Funding

The authors acknowledge funding received from the Department of Science and Technology of Sichuan Province and Southwest Medical School (2021ZYD0084, 2022NSFSC1381, 2021ZKZD006, and 2021ZKZD004).

## Conflict of interest

The authors declare that the research was conducted in the absence of any commercial or financial relationships that could be construed as a potential conflict of interest.

## Publisher’s note

All claims expressed in this article are solely those of the authors and do not necessarily represent those of their affiliated organizations, or those of the publisher, the editors and the reviewers. Any product that may be evaluated in this article, or claim that may be made by its manufacturer, is not guaranteed or endorsed by the publisher.

## Glossary

**Table:**

Aas	a hemagglutinin-autolysinadhesin
AC-3	adenylyl cyclase-3
Als	agglutinin-like sequence
AMPs	antimicrobial peptides
anti-MrpA	structural subunit of MR/P fimbriae
BECs	bladder epithelial cells;
bH/C	b-hemolysin/cytolysin
cAMP	cyclic adenosine monophosphate;
Candida.	Candida spp.
CCL20	C-C chemokine ligand 20
ClfA/B	Clumping Factors A and B
CNF1	cytotoxic necrotizing factor 1
COX-2	cyclooxygenase-2
CREB-1	cAMP-response element-binding protein-1;
CXCL1	C-X-C motif chemokine ligand 1
CXCL2	C-X-C motif chemokine ligand 2
CXCL5	C-X-C otif chemokine ligand 5
CX3CL1	CX3-C motif chemokine 1
CXCR4	CXC-motif chemokine receptor 4
DCs	dendritic cells
Ebp	endocarditis- and biofilm-associated
E. coli	Escherichia coli
E.f	Enterococcus faecalis
E.faecalis	Enterococcus faecalis
EGF	epidermal growth factor
EGFR	epidermal growth factor receptor
ERK	extracellular regulated protein kinases
Esp	enterococcal surface protein;
ExoU/T/S	exoenzyme U/T/S
F1C pili	type 1-like immunological group C pili
GBS	Group B Streptococcus
GLP-1	glucagon-like peptide-1
GM-CSF	granulocyte-macrophage colony-stimulating factor
GRIM-19	genes associated with retinoid-IFN-induced mortality-19
HA	Hyaluronic acid;
hBD	human b-defensin
HlyA	a-hemolysin
HMGN2	high-mobility group protein N2
HpmA	haemolysin
IBCs	intracellular bacterial communities;
LCN2	lipocalin-2
IDSA	Infectious Disease Society of America
IGF-1	insulin-like growth factors-1
IL-1	interleukin-1
IL-6	interleukin 6
IL-8	interleukin-8
INF-g	interferon-g
JNK	c-Jun-NH2-terminal kinase
K.p	Klebsiella pneumoniae
K. pneumoniae	Klebsiella pneumoniae
INF-g	interferon-g
LPS	lipopolysaccharide
MIP-1	macrophage inflammatory protein-1
MIP-1a	macrophage inflammatory protein-1a
MR/P pili	mannose-resistant Proteus-like
MyD88	myeloiddifferentiationfactor88;
NF-kB	Nuclear factor kappa beta
NK cells	natural killer cells
NLRP3	NOD-like receptor thermal protein domain associated protein 3
P.a	Pseudomonas aeruginosa
PAMP	pathogen-associated molecular pattern;
PAR2	Protease-activated receptor 2
PKA	protein kinase A
P.m	Proteus mirabilis
Pparg	peroxisome proliferator-activated receptor-g
P pili	pyelonephritis-associated pili
Pta	Proteus toxic agglutinin
PTX3	Pentraxins
QIRs	quiescent intracellular reservoirs
QS	quorum sensing;
RegIIIg	regenerating islet-derived 3g
RNase 7	ribonuclease 7
ROS	Reactive oxygen species
RSK	ribosomal s6 kinase
S.a	Staphylococcus aureus
SDF-1	stromal cell-derived factor1
SdrI	a surface-associated collagen-binding protein
SHH	sonic hedgehog
Siglec-9	sialic acid-binding Ig-like lectin-9;
SigV	extracytoplasmic function sigma factor
S.s	Staphylococcus saprophyticus
Ssp	a surface-associated lipase
SssF	S. saprophyticus surface protein F
Stat3	signal transducers and activators of transcription 3;
TcpF	TLR2-Toll/Interleukin-1 receptor domain-containing protein of E. Faecalis
TGF-a	transforming growth factor-a
THP	Tamm-Horsfall protein
TIR	TLR2-Toll/interleukin-1 receptor
TLR4	toll-like receptor 4;
TNF-a	tumor necrosis factor-a
TRPML3	transient receptor potential 3;
UafB	a cell wall-anchored protein
UPEC	Uropathogenic Escherichia coli;
UTI	Urinary tract infection
WHO	World Health Organization
ZapA	an extracellular metalloprotease, Mirabilysin.
